# Smart Dimmable LED Lighting Systems †

**DOI:** 10.3390/s22218523

**Published:** 2022-11-05

**Authors:** Milica Petkovic, Dragana Bajovic, Dejan Vukobratovic, Juraj Machaj, Peter Brida, Graeme McCutcheon, Lina Stankovic, Vladimir Stankovic

**Affiliations:** 1 Faculty of Technical Sciences, University of Novi Sad, 21000 Novi Sad, Serbia; 2 Faculty of Electrical Engineering and Information Technology, University of Zilina, 010 26 Zilina, Slovakia; 3 Ramboll, Clockwise, Glasgow G2 3BZ, UK; 4 Department of Electrical and Electronic Engineering, University of Strathclyde, Glasgow G1 1XQ, UK

**Keywords:** daylight, dimming, energy saving, illuminance design, users’ requirements, smart LED lighting systems

## Abstract

This paper proposes energy-efficient solutions for the smart light-emitting diode (LED) lighting system, which provides minimal energy consumption while simultaneously satisfying illuminance requirements of the users in a typical office space. In addition to artificial light from dimmable LED lamps, natural daylight coming from external sources, such as windows, is considered as a source of illumination in an indoor environment. In order to reduce total energy consumption, the smart LED system has the possibility to dim LED lamps, resulting in reduced LED output power. Additionally, various LED lamps’ functionality, such as semi-angle of the half illuminance and LED tilting, are introduced as an additional parameter to be optimized to achieve greater energy saving of the designed system. In order to properly exploit external lighting, the idea to reduce overall daylight intensity at a users’ location is realized by the option to dim the windows with a shading factor. Based on the users’ requirements for a minimal and desired level of illumination, the proposed optimization problems can be solved by implementing different optimization algorithms. The obtained solutions are able to give instructions to a smart LED system to manage and control system parameters (LEDs dimming levels, semi-angles of the half illuminance, orientation of LEDs, the shading factor) in order to design total illumination, which ensures minimal energy consumption and users’ satisfaction related to illuminance requirements.

## 1. Introduction

Based on recent studies on modern society and current lifestyle, people spend more than 70% of the day in indoor environments, especially in developed countries [1,2,3]. In indoor environments, such as offices and homes, the illumination is mostly provided by lighting systems based on light-emitting diodes (LEDs) due to long lifetime and good quality of light. According to many analysis and statistics, about 20% of global electric energy usage is used for lighting, with tendency to be increased to 40% in the future [4,5,6,7]. As modern commercial and industry markets aim to achieve energy savings as much as possible, the smart LED lighting systems have received attention in both research communities and industry as an efficient means of energy conservation. Offering a modern, persistent, and energy-efficient method of illumination, the smart lighting market is predicted to exceed 47 billion *$* in the next 10 years [8].

Besides minimal energy consumption, the smart LED systems aim to improve the comfort of users and satisfy their requirements related to level of illumination at a certain locations. For example, since people spend large part of the day in the offices, it is beneficial to ensure a comfortable environment during working hours in order to avoid poor performance as a result of an inappropriate level of light [9]. In practice, the design of overall illumination in indoor environments can be quite complicated and challenging since users’ demands for a certain level of light are very personal, subjective, and frequently changing. With the development of different indoor wireless technologies, modern Internet of Things (IoT) applications are able to provide the information about occupant preferences, presence, and current illumination levels, which can be beneficial for a smart LED systems architecture to perform illumination optimization and reduce energy consumption.

Many recent studies analyzed low energy efficiency and high user comfort within smart LED systems in different scenarios [10,11,12,13,14,15,16,17,18,19,20]. More precisely, refs. [10,11,12,13] considered the smart LED systems with optimal dimming feature, capable of controlling optical LED output power, with the aim to achieve maximal energy savings while satisfying users’ comfort at the same time. Furthermore, in addition to artificial LED light, the indoor environments are also illuminated by external sources of light from different objects, e.g., windows, resulting in daylight illumination. Since daylight represents an important part of the total illumination in most indoor spaces, it should also be taken into account during the design of smart LED systems. Optimization problems of reducing total energy consumption of smart LED systems with daylight taken into account were analyzed in [14,15,16,17,18]. A recent paper [21] studied cloud-based lighting control systems and proposed solutions to adapt from building- to human-centric environments by providing customized and automated indoor luminous environments.

Complete surveys of the smart lighting systems were presented in [19,20]. A detailed state-of-the-art of the smart lighting systems is given in [19], mostly focusing on industrial area. In addition, a detailed literature overview of the same topic [20] introduced a new machine learning application within smart lighting to improve user comfort.

Inspired by the aforementioned research, the aim of this paper is to establish an efficient strategy to reduce total energy consumption of the smart user-centric LED lighting system implemented in an indoor office space. The possibility to dim each LED lamp is introduced in order to control the artificial illumination and to provide energy savings. This way, illuminance contribution in the room can be managed by determined, optimized dimming levels of LEDs, while minimizing total energy consumption of the smart LED system, simultaneously satisfying users’ requirements for illumination. Furthermore, different functionalities of the LED lamp are identified as a potential parameter to be optimized in order to minimize energy consumption of the designed smart LED system and/or to satisfy users’ requirements for illumination. To the best of the authors’ knowledge, for the first time, the possibility to optimize the semi-angle at the half-illuminance of LED, and later the LED tilting in terms of LED orientation, are included. The expanded optimization problem results in more efficient managing of the total illuminance in the room, while providing better energy performance of the smart LED system. Additionally, the system model is augmented by considering daylight contribution from the windows. Since commercial tintable smart windows can be employed as a way to reduce and manage the daylight contribution in indoor space [22,23,24], the optimization problem is expanded with respect to shading factor, which is related to shading the windows and reducing the daylight contribution. Finally, after taking into account all optimization parameters, the total illuminance contribution can be managed and controlled by smart LED system in the most effective way to ensure the most convenient system performances.

The rest of the paper is organized as follows. Section 2 presents the system model together with the LED illumination model. Problem formulation is established in Section 3, together with numerical results. Section 4 considers a more general case when daylight is taken into account. Section 5 gives some concluding remarks.

## 2. Smart Dimmable LED Lighting System Model

**Figure 1:**
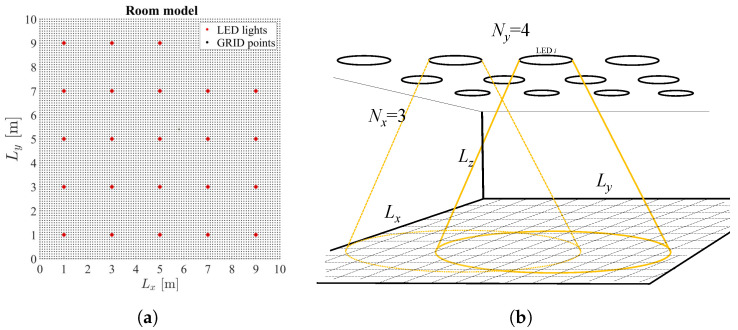
Considered system model: (**a**) the grid model of the ceiling with the LED lamp positions (red dots); (**b**) 3D indoor office environment.

The considered scenario assumes an indoor office environment with rectangular-shaped floor area of dimensions $L_{x}\times L_{y}$ meters and height $L_{z}$ meters. A smart LED lighting system with *N* ceiling-mounted LED lamps is employed to illuminate the indoor floor plane. In order to provide energy saving, dimmable LED lamps are implemented, resulting in an artificial LED illumination in the room that is controlled by adjusting dimming levels of each LED lamp. Dimmable ceiling-mounted LED lights are placed on a rectangular grid ($N=N_{x}\times N_{y}$) to illuminate the space, and their positions are considered to be fixed, and known to the smart LED system. Each LED lamp consists of *l* LEDs photodiodes [25]; thus, the maximum transmitted optical power of a LED lamp equals $P_{t}=lP_{l}$, where $P_{l}$ is the transmitted optical power of a single LED lamp. Each LED covers a certain circular coverage area on a floor plane. An overlapping beam scenario is considered where users can be covered by multiple LED beams. The floor plane is considered to be a horizontal plane divided into $M(M=M_{x}\times M_{y})$ grid points, each representing possible users’ locations. Figure 1a represents an example of the considered room model with LED lamps and grid point positions, while Figure 1b depicts the considered 3D indoor office environment with LEDs and possible user locations.

Since dimmable LED lamps are employed within the smart lighting system, the LED output power is related to the dimming vector s defined as:

(1) $$s={\left[s_{1},s_{2},\ldots ,s_{N}\right]}^{T},\;0\leq s_{i}\leq 1,\;i=1,2,\ldots ,N,$$

where $s_{i}$ represents the dimming level of the *i*-th LED lamp. The value $s_{i}=0$ results in turning off the *i*-th LED, while the value $s_{i}=1$ indicates that the *i*-th LED radiates at its maximum power $P_{t}$. Based on former, it follows that the output power of the *i*-th LED source is $P_{i}=s_{i}P_{t}$, where $P_{t}$ is the maximum LED power assumed to be the same for all LEDs in the considered system.

The illumination vector z, representing the illumination at the *M* points on the workspace plane, can be defined as as:

(2) $$z={\left[z_{1},\ldots ,z_{j},\ldots ,z_{M}\right]}^{T},\;j=1,2,\ldots ,M,$$

where $z_{j}$ is the illuminance at the *j*-th grid point.

First, it is assumed that the dimmable LED lighting system is the only source of light in the room, which leads to a simple linear model which relates the dimming vector s and the resulting illumination on the task plane points z as [10]:

(3) z=H·s,

where H represents the illuminance M×N matrix with element $h_{ji}\;(j=1,2,\ldots ,M,$ i=1,2,…,N) that determines the illuminance at the *j*-th point when the *i*-th LED is fully turned on ($s_{i}=1$), while all other LEDs are turned off $(s_{j}=0$ for j≠i,j=1,2,…,N). Clearly, the dimming vector s affects both the illumination and energy consumption of the LED system.

**Figure 2:**
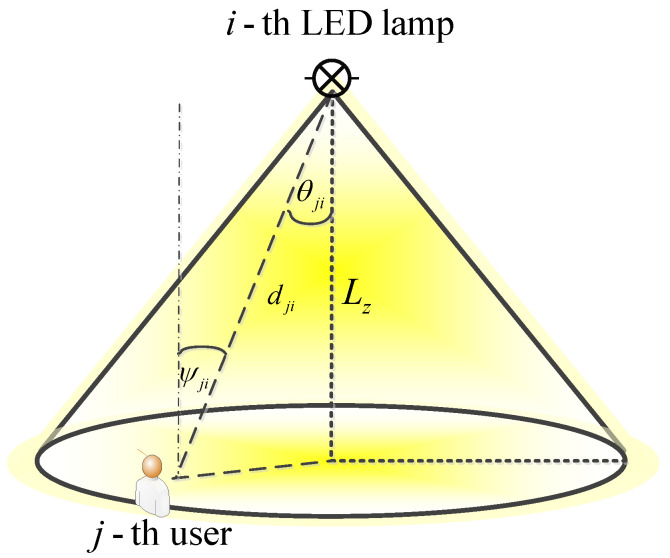
A LED illumination model.

LED illumination model: The illuminance at the *j*-th point when the *i*-th LED is set to maximum output power, while all other LEDs are turned off, is defined as [26,27,28]:

(4) $$h_{ji}=\frac{I\left(0\right)\cos^{m_{i}}\left(\theta_{ji}\right)}{d_{ji}^{2}}\cos\left(\psi_{ji}\right),$$

where $I\left(0\right)$ is the centre luminous intensity of the LED, $\theta_{ji}$ represents the angle of irradiance with respect to the axis normal to the ceiling, $\psi_{ji}$ is the angle of incidence with respect to the axis normal to the working plane surface, and $d_{ji}$ denotes the distance between the *i*-th LED and the *j*-th user position, as presented in Figure 2. The assumption that the LED and user plane surface are parallel is adopted; thus, $\theta_{ji}=\psi_{ji}$. LED lighting is described by a Lambertian radiation pattern with the order $m_{i}$ defined as [26,27,28]:

(5) $$m_{i}=-\frac{\ln2}{\ln\left(\cos\Phi_{1/2,i}\right)},$$

where $\Phi_{1/2,i}$ represents the semi-angle at the half-illuminance of the *i*-th LED. We consider that all LED lamps are characterized by the same semi-angle at the half-illuminance (i.e., $\Phi_{1/2,i}=\Phi_{1/2}$ and $m_{i}=m$ for all i=,1,⋯,N).

**Figure 3:**
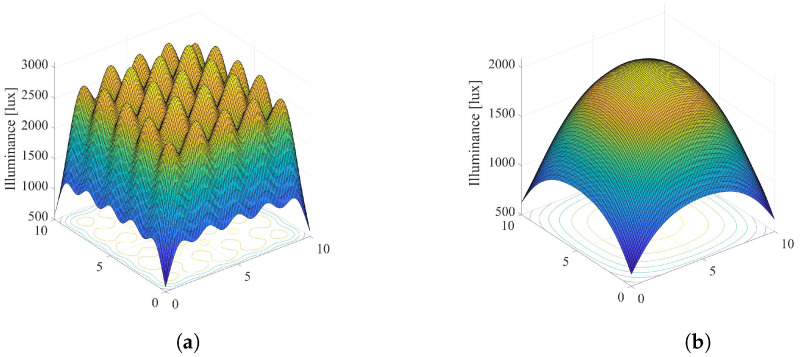
Overall illumination of the system without LEDs dimming, $s_{i}=1,\;i=1,\ldots ,N$. (**a**) $\Phi_{1/2}=20^{0}$. (**b**) $\Phi_{1/2}=60^{0}$.

**Figure 4:**
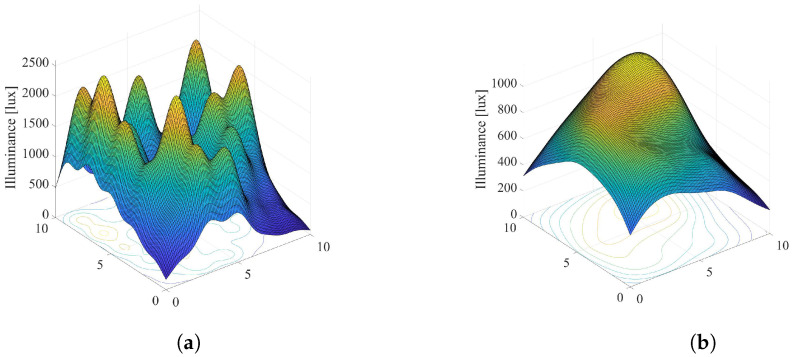
Overallillumination a randomly selected dimming vector, $0\leq s_{i}\leq 1,\;i=1,\ldots ,N$. (**a**) $\Phi_{1/2}=20^{0}$. (**b**) $\Phi_{1/2}=60^{0}$.

Overall illumination in the room: Figure 3 and Figure 4 present graphical examples of considered smart LED system applied to a typical office space with dimensions $L_{x}\times L_{y}\times L_{z}=10\times 10\times 3$ meters with N=25 dimmable ceiling-mounted LED lamps arranged as an $N_{x}\times N_{y}=5\times 5$ array. Each LED lamp consists of l=100 LEDs, arranged as an 10×10 array. The number of grid points on the horizontal floor plane is adopted to be M=100×100=10000. Moreover, we adopted the following model for LED output power, derived from the example presented in [26]. Input voltage of LED and input current are 6.42 V and 700 mA, respectively, resulting in the electrical power $P_{e}=4.494$ W. The electrical/optical conversion efficiency is 0.101, the optical output power of each LED is $P_{l}=0.452$ W, and the total luminous flux is Φ=107.16 lm. The values of LED system parameters defined above are adopted in the rest of the paper if not otherwise stated.

The centre luminous intensity of LED, $I\left(0\right)$, can be determined for different values of the semi-angle at the half-illuminance based on defintion of the total luminous flux Φ [26]

(6) $$\Phi =\frac{2\pi I\left(0\right)}{\left(1+m\right)}.$$

For constant total luminous flux (Φ=107.16 lm), based on (6), the centre luminous intensity of LED will be different for varios values of *m*, i.e., the semi-angle at the half-illuminance. The values of the parameter $I\left(0\right)$ and parameter *m* for diferent values of semi-angle at the half-illuminance are given in Table 1.

Taking all definitions and values of LED system parameters into account, overall illumination in the room is simulated and presented in Figure 3 and Figure 4. Figure 3 presents scenario when all LED lamps are set to the maximum output power ($s_{i}=1,\;i=1,\ldots ,N$), which results in maximal energy consumption. Additionally, adopted values of the semi-angle at the half-illuminance are $\Phi_{1/2}=20^{0}$ and $\Phi_{1/2}=60^{0}$ in Figure 3a,b, respectively. The semi-angle determines the wideness of the optical beam at the LED lamp output. Lower values of $\Phi_{1/2}$ results in narrower output optical beam. Figure 4 depicts the same system scenario, but for randomly selected dimming levels of LED sources. It is obvious that the dimming vector has significant impact on the overall distribution of the illumination in the indoor environment.

## 3. Problem Formulation and Results

The main aim of our work is to design the smart LED illumination system for *K* users residing in a horizontal workspace plane parallel to the ceiling. User devices are located at some of the previously defined grid points, thus their positions are known, based on an earlier estimation with any indoor positioning technique [29,30]. We tend to design an optimal LED illumination configuration which provides minimal energy consumption. In other words, the main purpose of our smart LED system is to design illumination vector z based on users’ requirements by selecting an optimized dimming vector s.

**Problem** **1.** *Formulation: Find an optimal energy consumption by dimming vector s that satisfies users’ illumination requirements at their locations.*

The adopted scenario considers two types of user illumination requirements: desired and minimal level of illumination. This means that the *j*-th user tends to be illuminated by a desired level of illumination $r_{j}$ (i.e., $z_{j}\approx r_{j}$), as well as that it requires that the level of illumination at its position is not lower than minimal requirement $l_{j}$ (i.e., $z_{j}\geq l_{j}$).

The vector of desired illuminance requirements for *K* users is defined as:

(7) $$r={\left[r_{1},\ldots ,r_{j},\ldots ,r_{K}\right]}^{T},\;j=1,2,\ldots ,K,$$

while the vector of minimal illuminance requirements for *K* users is defined as:

(8) $$l={\left[l_{1},\ldots ,l_{j},\ldots l_{K}\right]}^{T},\;j=1,2,\ldots ,K.$$

The optimal illumination vector z=H·s will clearly depend on definition of user requirements, which should be satisfied. Note that for considered scenario with *K* users, the size of vector z is now *K*, while the size of the illuminance matrix H is K×N.

Energy consumption of the smart LED system is related to the total system output power determined as $P_{t}\cdot \sum_{i=1}^{N}s_{i}$, where $P_{t}$ is the maximum transmitted optical power of a LED lamp previously defined as $P_{t}=lP_{l}$. Dimming vector s affects both illumination configuration and energy consumption, which means that the energy consumption of designed smart LED system is directly and linearly related to the $\ell_{1}$-norm of vector s, i.e., ${\left||s|\right|}_{1}=\sum_{i=1}^{N}s_{i}$.

In order to have insight into the energy saving contribution, the energy saving factor *G* is defined as a ratio between the total consumed power of the designed system and the total system output power when all LEDs are fully turned on, i.e.,

(9) $$G=\frac{P_{t}{\left||s|\right|}_{1}}{P_{t}N}=\frac{{\left||s|\right|}_{1}}{N}.$$

Favouring solutions with lower *G*, i.e., smaller ${\left||s|\right|}_{1}$, leads to reduced energy consumption.

In order to optimize dimming vector s, the optimization problem for a LED scenario that is considered to be smart is formulated as:

(10) $$\begin{array}{cc} \underset{s}{\mathrm{minimize}} & {∥s∥}_{1}+\lambda ∥H\cdot s-r∥\\ \mathrm{subject}\;\mathrm{to} & H\cdot s\geq l\\ \; & 0\leq s\leq 1,\;s\in R^{N} \end{array},$$

where parameter λ(0≤λ≤1) sets the priority by tuning the relative importance of the minimizing energy consumption and satisfying users’ requirements, and H represents the illuminance K×N matrix with elements $h_{ji}\;\left(j=1,2,\ldots ,K,\;i=1,2,\ldots ,N\right)$ defined in (4). The optimization proposed problem in (10) can be efficiently solved in software package MATLAB.

**Figure 5:**
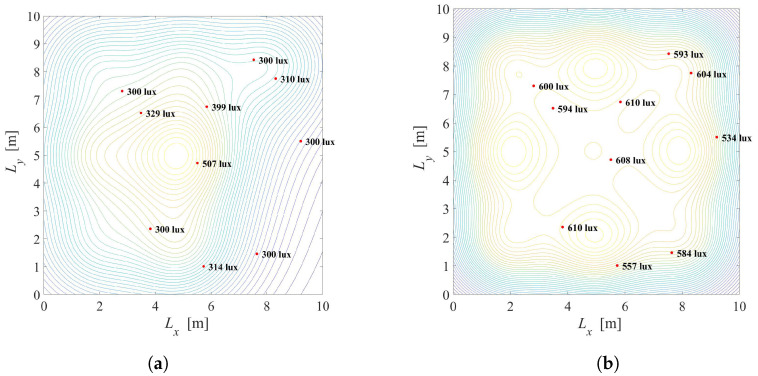
Solution of the Problem 1 defined in (10). (**a**) λ=0.01, G=0.44, $\Phi_{1/2}=60^{0}$. (**b**) λ=0.5, G=0.92, $\Phi_{1/2}=60^{0}$.

Results: Figure 5 depicts the illumination distribution when K=10 users with known positions are placed randomly in the office. The semi-angle at half-illuminance is $\Phi_{1/2}=60^{0}$, and smart LED system employs N=3×3 LED lamps. Desired and minimal illuminance requirements are the same for all users and equal to $r_{j}=600$ and $l_{j}=300$ lux for j=1,2,…,K, respectively. In Figure 5a, the priority parameter is λ=0.01 meaning that the primary task is to reduce total energy consumption. Two of nine LEDs are turned off, while seven LEDs are turned on and dimmed to give a certain level of illumination with maximal possible energy savings. The energy saving factor is G=0.44 for this scenario. The illumination distribution of the same system with λ=0.5 is presented in Figure 5b, pointing that the satisfaction of the users’ requirements has priority over energy savings. In this case, even five of nine LEDs are fully turned on and the energy saving factor is G=0.92.

Based on the results presented in Figure 5a,b, it can be concluded that *G* is significantly higher in Figure 5b resulting in more power consumption. Still, the level of illumination on users’ position is close to the desired level, so the users’ illumination requirements will be fulfilled to great extent. By tuning the priority parameter λ, the compromise between energy saving and satisfaction of illumination requirements can be achieved.

### 3.1. Effect of the Optical Signals Reflections

The previous illumination model is based on the direct Line of Sight (LoS) propagation model between LEDs and users’ devices. Still, optical signals will be reflected from surrounding surfaces in indoor environments, which will have an impact on the overall illumination in the room. From the *i*-th LED lamp, light can reach the *j*-th grid point after number of reflections. In order to apply a more general illumination model that takes into account both LoS and diffuse reflection components, we adopt a recursive method presented in [31].

After multiple reflections, the illumination at the *j*-th grid point from the fully turned on *i*-th LED lamp (while all other LEDs are turned off) can be expressed as [31]

(11) $$h_{ji}=\sum_{k=0}^{\infty }h_{ji}^{\left(k\right)},$$

where $h_{ji}^{\left(k\right)}$ is the illumination component after exactly *k* reflections.

When k=0, i.e., for direct LoS component at the *j*-th grid point from the *i*-th LED lamp, the illuminance component was previously defined in (4) as:

(12) $$h_{ji}^{\left(0\right)}=\frac{I\left(0\right)\cos^{m}\left(\theta_{ji}\right)}{\rho_{ji}^{2}}\cos\left(\psi_{ji}\right).$$

For k>0, the illumination component $h_{ji}^{\left(k\right)}$ can be determined recursively as:

(13) $$h_{ji}^{\left(k\right)}=\int_{i}\rho_{ref}h_{ji}^{\left(0\right)}*h_{ji}^{\left(k-1\right)},$$

where the symbol ∗ represents convolution and $\rho_{ref}$ is the reflection coefficient dependent on the surface material. Note that, for the *k*-th reflection, the lighting source is the receiving point of the previous (k−1)-th reflection, and so on.

**Figure 6:**
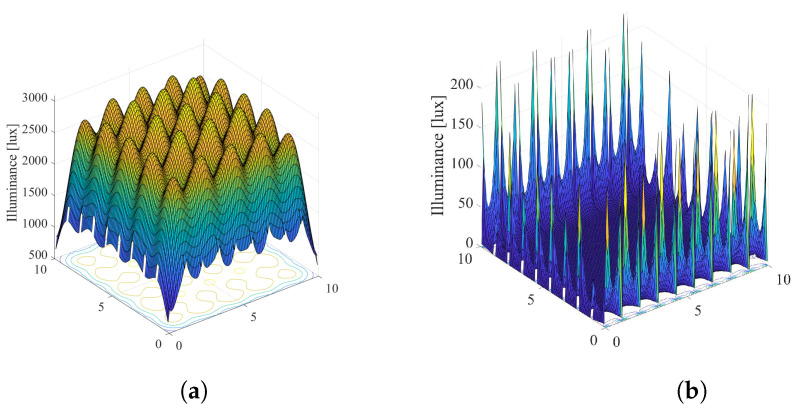
Overall illumination when $s_{i}=1\;\forall i$, $\Phi_{1/2}=20^{0}$. (**a**) Joint illumination for k=0 and k=1. (**b**) Illumination only for k=1.

**Figure 7:**
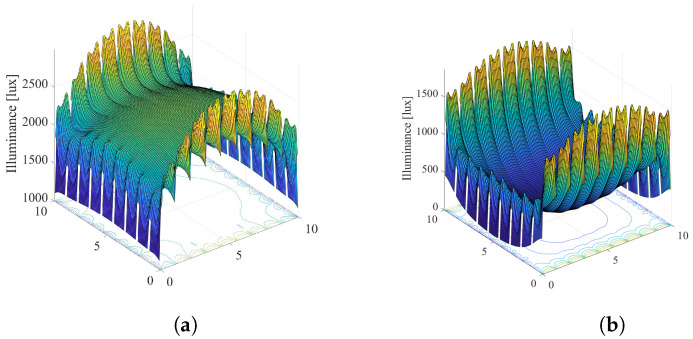
Overall illumination when $s_{i}=1\;\forall i$, $\Phi_{1/2}=60^{0}$. (**a**) Joint illumination for k=0 and k=1. (**b**) Illumination only for k=1.

Based on the presented recursive method [31], we simulated the overall illumination in the room by taking into account the direct LoS component (k=0) and the first reflected component (k=1) from the walls, i.e., only the first two terms of the summation in (11). Other reflections are ignored. The reflection coefficient takes a value $\rho_{ref}=0.8$. For the same system configuration as in Figure 3, the overall illumination in the room considering k=0 and k=1 components, as well as the illumination only from k=1 component, is presented in Figure 6 and Figure 7, when the semi-angle is $\Phi_{1/2}=20^{0}$ and $\Phi_{1/2}=60^{0}$, respectively. From the presented results, it can be concluded that that the first reflection has important impact only near the walls in the indoor environments, and for higher values of the semi-angle.

**Figure 8:**
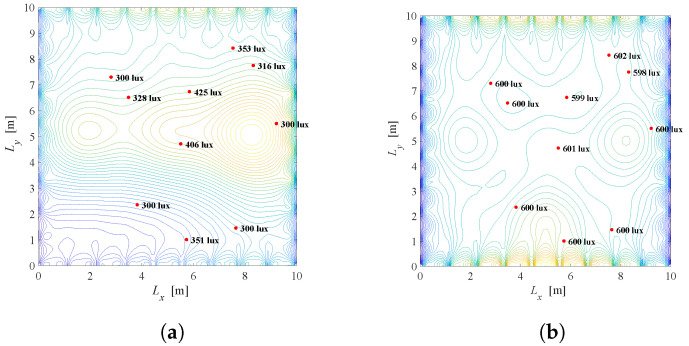
The solution of the Problem 1 defined in (10) while taking into account the direct LoS and the first reflection. (**a**) λ=0.01, G=0.36, $\Phi_{1/2}=60^{0}$. (**b**) λ=0.5, G=0.75, $\Phi_{1/2}=60^{0}$.

Next, the same problem defined in (10) is solved, but the illuminance matrix H is determined by the elements $h_{ji}\;\left(j=1,2,\ldots ,K,\;i=1,2,\ldots ,N\right)$ defined in (11) while taking only the first two terms of the summations into account (direct (k=0) and the first reflected component (k=1)). Comparing results from Figure 5 and Figure 8, it can be concluded that the first reflection reduces the energy saving factor *G* and that contributes to the energy consumption to a some extent. Still, the main conclusion is not changed if the illumination model is based only on the direct LoS components; thus, in the following analysis, we will ignore the reflections.

### 3.2. The Semi-Angle at the Half-Illuminance of Optimization Problem

In order to minimize energy consumption and satisfy illuminance requirements, optimization problem in (10) can be expanded by considering the semi-angle at the half-illuminance of LEDs. Using beam-shaping lenses or different co-centric LEDs, the smart lightning system can implement the LEDs with tunable semi-angles.

With the assumption that lenses implemented at each LED transmitter can be managed, the semi-angle at the half-illuminance can take several discrete values (e.g., 20${}^{\circ }$, 40${}^{\circ }$, 60${}^{\circ }$ or 80${}^{\circ }$), i.e.,

(14) $$\Phi_{1/2}=\left[\phi_{1},\phi_{2},\ldots ,\phi_{N}\right],\;\phi_{i}\in \left\{20^{\circ },40^{\circ },60^{\circ },80^{\circ }\right\},\;i=1,2,\ldots ,N.$$

Recall that the semi-angle at the half-illuminance affects the order *m* of a Lambertian radiation pattern and the centre luminous intensity of LED, $I\left(0\right)$ (see (5) and (6)). For constant total luminous flux Φ=107.16 lm, the values of the parameters $I\left(0\right)$ and *m* for different values of semi-angle can be found in Table 1.

**Problem** **2.** *Formulation: Minimise energy consumption by dimming vector s and the semi-angle $\Phi_{1/2}$ while satisfying users’ illumination requirements at their locations.*

If we consider the illumination propagation model in (4), the value of the semi-angle affects both the order *m* and the centre luminous intensity and thus has impact on the illuminance matrix H. In order to optimize both dimming vector s and semi-angle $\Phi_{1/2}$, the optimization problem becomes discrete and non-convex:

(15) $$\begin{array}{cc} \underset{s,\Phi_{1/2}}{\mathrm{minimize}} & {∥s∥}_{1}+\lambda ∥H\cdot s-r∥\\ \mathrm{subject}\;\mathrm{to} & H\cdot s\geq l\\ \; & 0\leq s\leq 1,\;s\in R^{N}\\ \; & 0\leq \Phi_{1/2}\leq \pi /2 \end{array}.$$

For a limited number of discrete semi-angles, the optimization problem in (15) can be solved in MATLAB.

**Figure 9:**
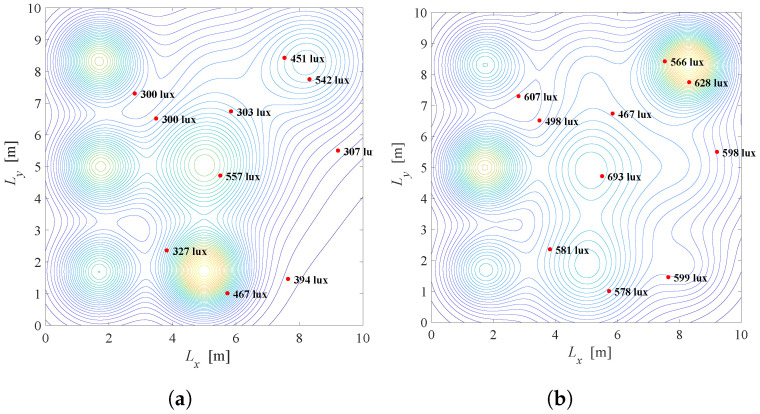
Solution of the Problem 2 defined in (15). (**a**) λ=0.01, G = 0.31. (**b**) λ=0.5, G = 0.61.

Results: Figure 9 shows the illumination distribution when K=10 and the smart LED system employs N=3×3 LED lamps, of the optimisation solution of (15), i.e., when the discrete semi-angle values for each LED are $\phi_{i}\in \left\{20^{\circ },40^{\circ },60^{\circ }\right\}$. In order to see if there is a benefit in expanding the optimization problem, we compare Figure 5 and Figure 9. Note that both figures show the results with the same scenario, only with and without possibility to optimze the semi-angle is added. Comparing results in Figure 5a and Figure 9a, when λ=0.01 meaning that the primary task is energy saving, it can be concluded that energy saving factor *G* is reduced from G=0.44 to G=0.31. From Figure 5b and Figure 9b it can be observed that a greater reduction of energy consumption is achieved when the priority parameter λ=0.5 since the energy saving factor *G* is reduced from G=0.92 to G=0.61.

Based on the results presented in Figure 5 and Figure 9, it can be concluded that a significant energy saving can be achieved by introducing the option of multiple semi-angle choices, at the expense of the system complexity due to employment of the beam-shaping lenses or different co-centric LEDs.

### 3.3. Led Tilting

With aim to further reduce energy consumption and/or to satisfy illuminance requirements, the optimization problem can be posed in terms of LED lamps tilting. It is assumed that each LED can be tilted by a certain angle in the room coordinate system. The orientation of the LED tilt in 3D is determined by two angles: the zenith angle, ρ, and the azimuth angle, ε. The zenith angle ρ (−π/2≤ρ≤π/2) corresponds to LED deviation in relation to the z-plane. The azimuth angle (0≤ε≤2π) represents the deviation in relation to the x-plane, i.e., it is the angle between the positive part of the x-axis and the projection on the x-y plane.

**Figure 10:**
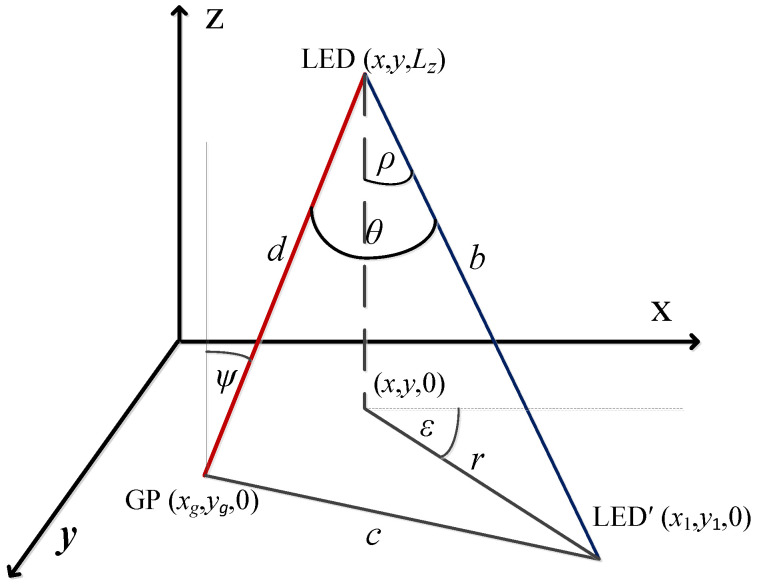
LED rotation coordinates.

If the LED lamp coordinates (The indexes *i* and *j* are omitted since the presented analysis is general and valid for all pairs of LED and grid points.) are LED (x,y,z) (note that $z=L_{z}$ is the height), the projection of the LED on x-y plane is given as LED (x,y,0). If it is assumed that the LED is tilted by the angles −π/2≤ρ≤π/2 and 0≤ε≤2π, the projection of the rotated LED axis (the height of the cone of LED lighting) on x-y plane is LED′ $\left(x_{1},y_{1},0\right)$. The distance between LED and LED′ on x-y plane is determined as $r=L_{z}\tan\left(\rho \right)$ (see Figure 10).

Since the coordinates of LED locations (x,y,0) are known, the coordinates LED′ $\left(x_{1},y_{1},0\right)$ can be determined as:

(16) $$\begin{array}{cc} \; & x_{1}=x+L_{z}\tan\left(\rho \right)\cos\left(\varepsilon \right),\\ \; & y_{1}=y+L_{z}\tan\left(\rho \right)\sin\left(\varepsilon \right). \end{array}$$

Note that the grid point coordinate GP $\left(x_{g},y_{g},0\right)$ are also known. Finally, based on the geometry of the system setup presented in Figure 10, the angle of irradiance can be determined as:

(17) $$\begin{array}{cc} \; & \cos\left(\theta \right)=\frac{d^{2}+b^{2}-c^{2}}{2db},\;\mathrm{for}\;c^{2}>d^{2}+b^{2}\;\left(\theta \leq \pi /2\right),\\ \; & d=\sqrt{L_{z}^{2}+{\left(x_{g}-x\right)}^{2}+{\left(y_{g}-y\right)}^{2}},\\ \; & b=\sqrt{L_{z}^{2}+{\left(x_{1}-x\right)}^{2}+{\left(y_{1}-y\right)}^{2}},\\ \; & c=\sqrt{{\left(x_{1}-x_{g}\right)}^{2}+{\left(y_{1}-y_{g}\right)}^{2}}. \end{array}$$

If the LEDs tilting is implemented within the smart lighting system, then the elements $h_{ji}$ of the illuminance matrix H are defined in (4), but the cosine of the angle of irradiance, $\cos\left(\theta_{ji}\right)$, is determined by (17), while the angle of incidence, $\psi_{ji}$, is related to the receiver characteristics and can be determined as:

(18) $$\cos\left(\psi_{ji}\right)=\frac{L_{z}}{d_{ji}},$$

where $d_{ji}$ is defined in (17).

**Figure 11:**
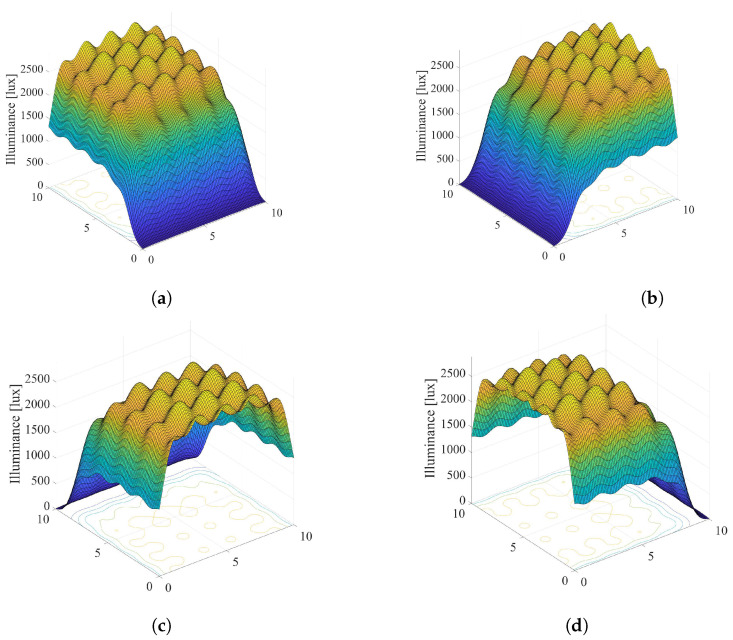
Overall illumination with LEDs tilting for $s_{i}=1\;\forall i$, $\Phi_{1/2}=20^{0}$, $\rho =30^{0}$. (**a**) $\rho =30^{0}$, $\varepsilon =0^{0}$. (**b**) $\rho =30^{0}$, $\varepsilon =90^{0}$. (**c**) $\rho =30^{0}$, $\varepsilon =180^{0}$. (**d**) $\rho =30^{0}$, $\varepsilon =270^{0}$.

**Figure 12:**
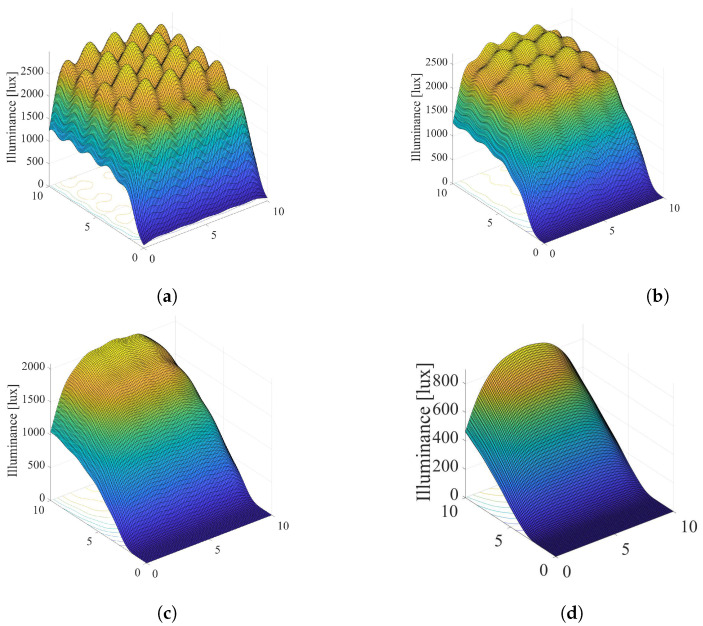
Overall illumination with LEDs tilting for $s_{i}=1\;\forall i$, $\Phi_{1/2}=20^{0}$, $\varepsilon =0^{0}$. (**a**) $\rho =20^{0}$, $\varepsilon =0^{0}$. (**b**) $\rho =40^{0}$, $\varepsilon =0^{0}$. (**c**) $\rho =60^{0}$, $\varepsilon =0^{0}$. (**d**) $\rho =80^{0}$, $\varepsilon =0^{0}$.

The overall illumination for the systems with the same configuration as in Figure 3a (without LEDs dimming, the semi-angle is $\Phi_{1/2}=20^{0}$) is presented in Figure 11 and Figure 12, considering different zenith and azimuth angels. Figure 11 represents the overall illumination in the room, for constant zenith angle, $\rho =30^{0}$, while the azimuth angle is $\varepsilon =0^{0}$, $\varepsilon =90^{0}$, $\varepsilon =180^{0}$ and $\varepsilon =270^{0}$. On the other hand, Figure 12 shows the overall illumination for constant azimuth angle, $\varepsilon =90^{0}$, while the zenith angle is $\rho =20^{0}$, $\rho =40^{0}$, $\rho =60^{0}$ and $\rho =80^{0}$. Based on the presented results, it is proved that the geometric analysis of tilted LED system is correct and affects overall illumination in the indoor space.

**Problem** **3.** *Formulation: Minimise energy consumption by dimming vector s and the tilting angles of LEDs, while satisfying user illumination requirements.*

By introducing the possibility to tilt the LEDs, the optimization problem in (10) can be expanded by considering different LED orientations. Since the orientation of the LEDs will affect the illuminace matrix H, the optimization problem is now updated as:

(19) $$\begin{array}{cc} \underset{s,(\rho ,\varepsilon )}{\mathrm{minimize}} & {∥s∥}_{1}+\lambda ∥H\cdot s-r∥\\ \mathrm{subject}\;\mathrm{to} & H\cdot s\geq l\\ \; & 0\leq s\leq 1,\;s\in R^{N}\\ \; & -\pi /2\leq \rho \leq \pi /2\\ \; & 0/2\leq \varepsilon \leq 2\pi \end{array}.$$

(19) can be solved using MATLAB.

**Figure 13:**
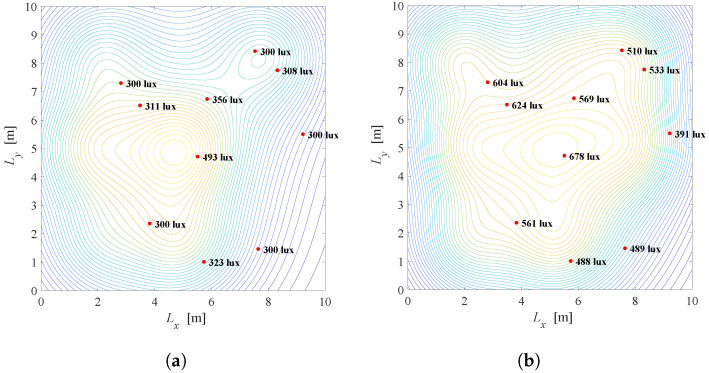
Solution of the Problem 3 defined in (19). (**a**) λ=0.01, G = 0.42. (**b**) λ=0.5, G = 0.79.

Results: Figure 13 presents the illumination distribution obtained by the solution of (19) for the same system configuration as in Figure 5. In the presented example, we considered three possible orientations of LEDs, defined by the pairs of the zenith and azimuth angles as $\left(\rho_{i},\varepsilon_{i}\right)\in \left\{\left(0,0\right),\left(30,0\right),\left(30,180\right)\right\}$. Based on the results in Figure 5a for the system without LED tilting, and results in Figure 9a for the system when LED tilting is employed, it can be concluded that energy saving can be achieved by introducing the possibility of different LEDs orientations. The energy saving is greater when λ=0.5, which means that the LEDs tilting can provide energy saving while satisfying users’ requirements. Note that the significant energy savings can be obtained with adding more options for LEDs orientation (this example considered only 3 options due to implemented optimization algorithm complexity), but at the expense of the system design complexity.

## 4. Effects of External Daylight in Smart Dimmable LED Lighting Systems

The model analysed above includes only artificial light in the indoor environment since the dimmable LED system is the only source of light. Still, the external sources of light (e.g., windows) usually cannot be ignored since natural daylight can significantly contribute to overall illumination in the room. If there are *K* users in the room located at some of the previously defined *M* grid points, illumination due to external sources at the users’ positions on the workspace plane, usually called daylight, is defined as:

(20) $$w={\left[w_{1},\ldots ,w_{j},\ldots ,w_{K}\right]}^{T},\;j=1,2,\ldots ,K,$$

where $w_{j}$ represents the total daylight intensity received at the *j*-th user device’s position. Note that daylight contribution is independent of the dimming LED vector s. With the aim to minimise energy consumption and to ensure satisfaction of the users’ requirements, smart home can employ a smart LED system by exploiting both artificial light and daylight. A smart home environment usually employs “smart windows” [22], which can manage the level of daylight intensity in the room. The windows can be uniformly shaded by a constant factor denoted by *a*(0≤a≤1) [23,24], e.g., via controllable blinds. When the shading factor is a=1, the windows are completely ‘open’ (blinds are off) and overall daylight intensity is present at the users’ location. In contrast, when a=0, windows are totally shaded, then the daylight will be absent (w=0).

Considering the general case when both artificial light and daylight are exploited, the illumination model in (3) can be easily updated with daylight distribution, resulting in the following illumination for the users:

(21) z=H·s+a·w.

**Problem** **4.** *Formulation: Find the minimum energy consumption by dimming vector s and shading factor a that satisfies users’ illumination requirements at their locations.*

Besides dimming vector s, the shading coefficient *a* can be also optimized in order to reduce the daylight distribution density over the room. In this way, it is possible to manage overall illumination through the open office space in order to reduce energy consumption in larger extent and/or to satisfy users’ demands. The newly formulated optimization problem is defined as:

(22) $$\begin{array}{cc} \underset{s,a}{\mathrm{minimize}} & {∥s∥}_{1}+\lambda ∥H\cdot s+a\cdot w-r∥\\ \mathrm{subject}\;\mathrm{to} & H\cdot s+a\cdot w\geq l\\ \; & 0\leq s\leq 1,\;s\in R^{N}\\ \; & 0\leq a\leq 1,\;a\in R \end{array},$$

where H is the illuminance matrix of size K×N, with elements given by (4). Priority parameter λ(0≤λ≤1) is previously defined as a way to manage a relative importance of the minimizing energy consumption and satisfying users’ requirements. To solve the optimization problem in (22), we can employ CVX, a MATLAB package for specifying and solving convex programs [32].

**Figure 14:**
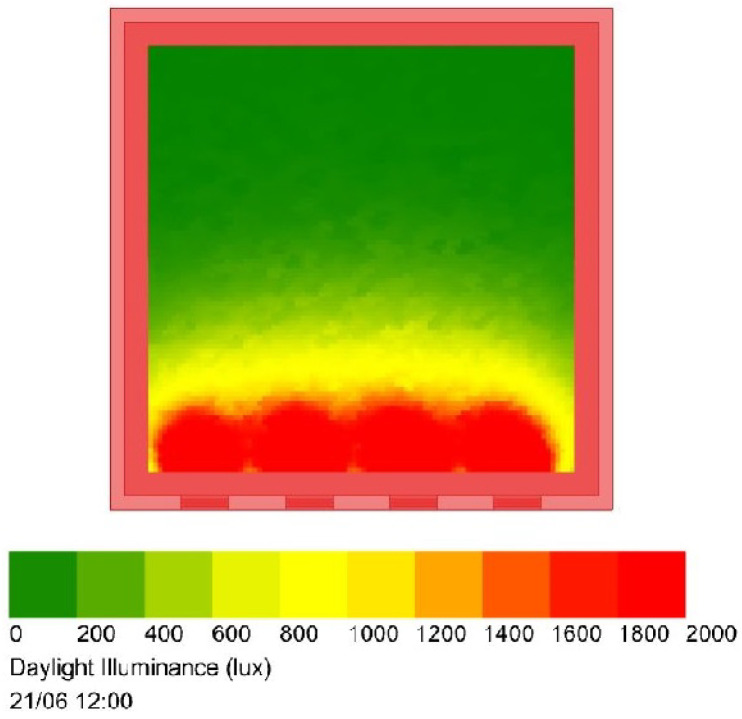
Daylight intensity in the considered office. The windows are positioned at the bottom of the figure [33].

Results: Simulation results are obtained for the example of a typical office with four windows, each with dimensions 1×2 m. While all LEDs are turned off, i.e., artificial lights are absent, measurements can be performed during different periods of the day in order to collect data about the level of daylight illuminance at the horizontal plane. This kind of measurement can be performed by employing different light sensor devices. Figure 14 presents the level of the daylight intensity which are obtained by measurements in June, at noon, in Glasgow, UK.

**Figure 15:**
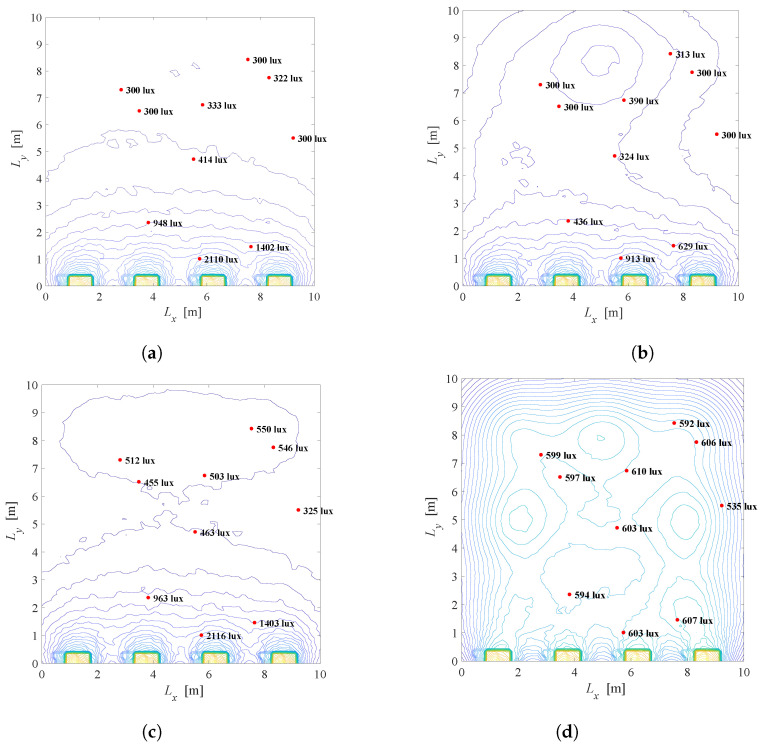
Solution of the Problem 4 defined in (22), K=10 users randomly located, $\Phi_{1/2}=60^{0}$. (**a**) λ=0.01, a=1, G = 0.17. (**b**) λ=0.01, a=0.42, G = 0.26. (**c**) λ=0.5, a=1, G = 0.33. (**d**) λ=0.5, a=0.09, G = 0.86.

For the same system setup as in Figure 5 ($K=10,N=3\times 3,\;\Phi_{1/2}=60^{0},$$r_{j}=600$ lux and $l_{j}=300$ lux for j=1,2,…,K), Figure 15 presents the illumination distribution based on the solution of the problem defined in (22). Figure 15a,b show the scenario when λ=0.01 and the priority is to achieve the energy savings. Figure 15a presents the system when the windows shading is not possible (a=1), while Figure 15b corresponds to the case when smart windows are employed. Although greater energy consumption is present in Figure 15b, users near the windows are illuminated with lower light intensity when the shading of the windows is available. For example, when a=1, a complete contribution of the light from the windows will affect users near windows, i.e., 2110 lux, 1402 lux, etc., due to strong component of daylight illumination close to windows. A similar conclusion can be taken from Figure 15c,d when λ=0.5. In both cases, the window shading is beneficial since it can achieve a better level of user satisfaction while maintaining energy savings.

**Figure 16:**
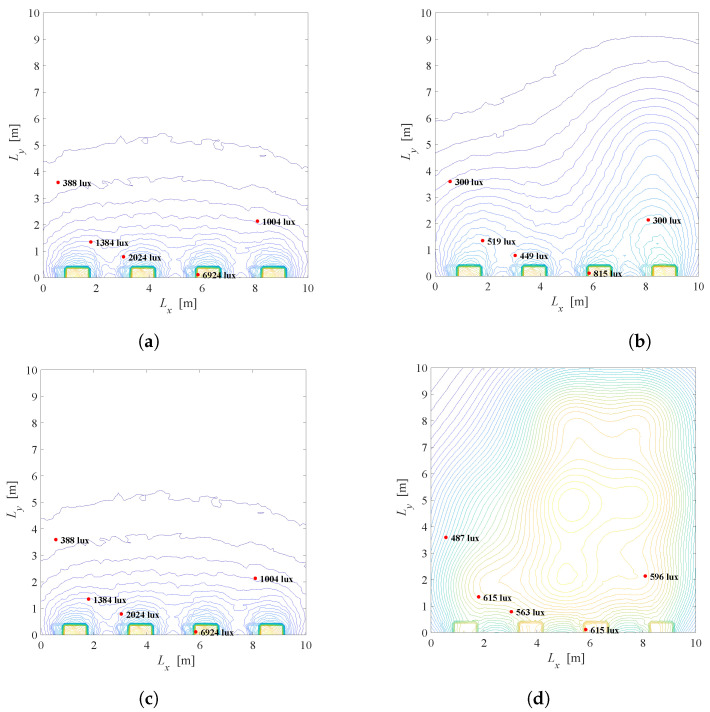
Solution of the Problem 4 defined in (22), K=5 users located near windows, $\Phi_{1/2}=60^{0}$. (**a**) λ=0.01, a=1, G ≈ 0. (**b**) λ=0.01, a=0.1, G = 0.22. (**c**) λ=0.5, a=1, G ≈ 0. (**d**) λ=0.5, a=0.03, G = 0.82.

**Figure 17:**
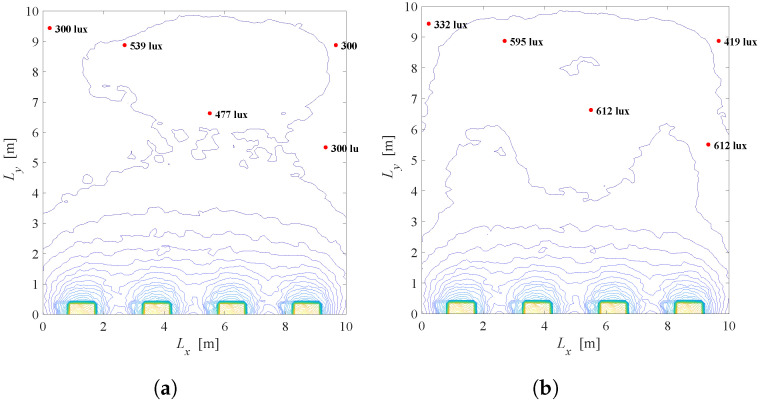
Solution of the Problem 4 defined in (22), K=5 users located opposite of the windows, $\Phi_{1/2}=60^{0}$. (**a**) λ=0.01, a=1, G = 0.32. (**b**) λ=0.5, a=1, G = 0.55.

A similar situation is presented in Figure 16, but for K=5 users located near the windows, for λ=0.1 and λ=0.5. If there is no possibility to shade the windows, users near the window will be illuminated with a significant level of light, which can be undesirable, resulting in inadequate seating positions. In contrast, a situation when K=5 users are placed opposite the windows is shown in Figure 17. Note that for the position of the users’ devices in Figure 17, after solving the optimization problem in (22), it is obtained that a=1, which is justified with the daylight distribution in Figure 14, as the daylight from the windows will have a minor effect in that part of the room. It is obvious that daylight from the windows should be exploited in order to reduce artificial LED lighting, but it is also important to take into consideration both minimal and desired users’ requirements for illumination.

**Figure 18:**
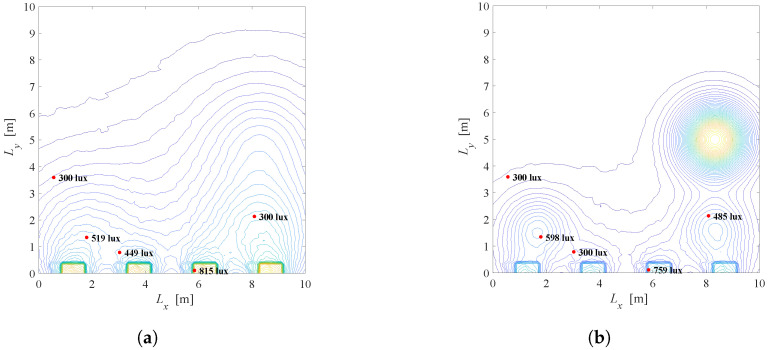
Solution of the Problem 4 defined in (22) for different semi-angles, λ=0.01. (**a**) $\Phi_{1/2}=60^{0}$, a=0.1, G = 0.22. (**b**) $\Phi_{1/2}=20^{0}$, a=0.1, G = 0.14.

Figure 18 observes the same problem, but in the context of the semi-angle of the half illuminance. As can be seen here, different semi-angle will have an impact on the overall illumination and energy saving, and thus should be considered as an important part of the smart LED systems, which is the topic of the next optimization problem.

### 4.1. Simultaneously Optimising Shading Window Factor and Semi-Angle at the Half-Illuminance

Similarly to Section 3.2, we will extend the optimization problem presented in (22) by introducing a possibility to optimize the semi-angle at the half-illuminance in order to minimize energy consumption.

**Problem** **5.** *Formulation: Minimise energy consumption by dimming vector s, shading factor a and the semi-angle $\Phi_{1/2}$ that satisfy users’ illumination requirements at their locations.*

Considering the problem posed in Section 3.2 (i.e., (15)), the optimization problem in (22) can be updated as follows:

(23) $$\begin{array}{cc} \underset{s,a,\Phi_{1/2}}{\mathrm{minimize}} & {∥s∥}_{1}+\lambda ∥H\cdot s+a\cdot w-r∥\\ \mathrm{subject}\;\mathrm{to} & H\cdot s+a\cdot w\geq l\\ \; & 0\leq s\leq 1,\;s\in R^{N}\\ \; & 0\leq a\leq 1,\;a\in R\\ \; & 0\leq \Phi_{1/2}\leq \pi /2 \end{array},$$

where different values of $\Phi_{1/2}$ result in different order *m* and in different centre luminous intensity, i.e., different illuminace matrix H. This optimization problem is solved in MATLAB with a help of a package CVX [32].

**Figure 19:**
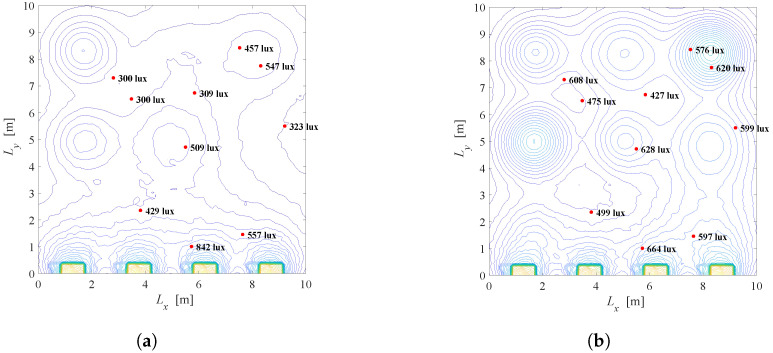
Solution of the Problem 5 defined in (23). (**a**) λ=0.01, a=0.39, G = 0.19. (**b**) λ=0.5, a=0.29, G = 0.52.

Results: The system configuration from analysis in Section 3.2 (Figure 9) is extended by considering daylight sources as it was previously discussed, i.e., daylight contribution is defined by Figure 14. Recall that the discrete semi-angle values for each LED are $\phi_{i}\in \left\{20^{\circ },40^{\circ },60^{\circ }\right\}$. Comparison of Figure 15b and Figure 19a for λ=0.01, i.e., Figure 15d and Figure 19b for λ=0.5, leads to a conclusion that introducing the extra component for optimization results in greater energy saving while satisfying users’ requirements. For example, when the achieved energy saving factor in Figure 15b is G=0.26, it is G=0.19 in Figure 19a. Introducing the selection of an optimal value of $\Phi_{1/2}$ results in important minimization of energy consumption. This will be more pronounced if the smart LED system has a larger set of discrete values of semi-angles as a choice.

### 4.2. Simultaneously Optimising Shading Window Factor and LED Tilting

As additional component, the orientation of LED lamps is added as optimization parameter to the problem defined in (22). Based on analysis presented in Section 3.3, the optimization problem is defined as follows.

**Problem** **6.** *Formulation: Find dimming vector s, shading factor a and tilting angles of LEDs that simultaneously minimise energy consumption and satisfies users’ illumination requirements at their locations.*

Based on the problem observed in Section 3.3 (i.e., (19)), the optimization problem in (22) is now defined as:

(24) $$\begin{array}{cc} \underset{s,a,(\rho ,\varepsilon )}{\mathrm{minimize}} & {∥s∥}_{1}+\lambda ∥H\cdot s+a\cdot w-r∥\\ \mathrm{subject}\;\mathrm{to} & H\cdot s+a\cdot w\geq l\\ \; & 0\leq s\leq 1,\;s\in R^{N}\\ \; & 0\leq a\leq 1,\;a\in R\\ \; & -\pi /2\leq \rho \leq \pi /2\\ \; & 0/2\leq \varepsilon \leq 2\pi \end{array},$$

where previously defined azimuth and zenith angles, i.e., (ε) and (ρ), respectively, have impact on the illumination matrix H.

**Figure 20:**
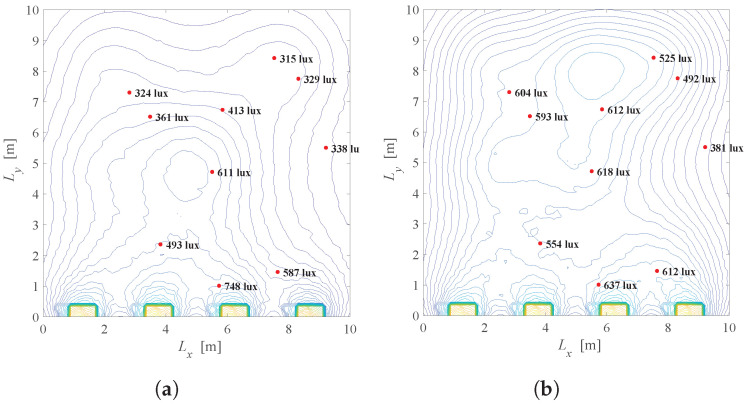
Solution of the Problem 6 defined in (24). (**a**) λ=0.01, a=0.2, G = 0.2; (**b**) λ=0.5, a=0.16, G = 0.73.

Results: After adding the daylight contribution presented in Figure 14 to the system configuration from Section 3.3 (Figure 13), problem defined in (24) is solved using MATLAB and CVX package [32]. Three possible orientations of LEDs, defined by the pairs of the zenith and azimuth angles as $\left(\rho_{i},\varepsilon_{i}\right)\in \left\{\left(0,0\right),\left(30,0\right),\left(30,180\right)\right\}$, are considered. Results are presented in Figure 20. In order to observe benefits of possible LEDs orientations, we will compare Figure 15b and Figure 20a for λ=0.01, and Figure 15d and Figure 20b for λ=0.5. Greater energy saving is noticed with introducing the LED tilting in the smart LED system, which further can be improved with a greater number of options for LEDs orientation, but at the expense of the system complexity and costs.

## 5. Conclusions

In this paper, we have proposed an energy-efficient strategy for the smart lighting system. In order to provide minimal energy consumption and/or satisfy users’ requirements for illumination level, the smart LED system can control and manage the overall illumination in the indoor space by considering that the LED lamps have the possibility to be dimmed. Furthermore, in addition to artificial LED light, external sources of light have also been included. With the aim to properly exploit external lighting while providing minimal energy consumption, we have proposed that the levels of daylight are managed by window shading. As an additional parameter to be optimized in order to improve performance of the designed smart lighting system, we have observed both the semi-angle of the half illuminance and LED orientations. After solving the optimization problems by implementing algorithm in MATLAB software, it has been proved by a sequence of illustrative examples that significant energy savings can be achieved while simultaneously satisfying users’ demands. It has been proved that different combinations of system parameters can lead to significant energy saving, and thus various solutions of the optimization problems can be beneficial to design an efficient smart LED system. Since implementation of different smart LED system setups can be more complex with the introduction of new optimization parameters, designers should pay attention to the trade off between system complexity, efficiency, and users’ requirements.

## Figures and Tables

**Table 1:** Values of *m* and $I\left(0\right)$ based on semi-angle at the half-illuminance, Φ=107.16 lm.

$\Phi_{1/2}{[}^{0}]$	*m*	$I\left(0\right)$ [cd]
10	45.27	789.3
20	11.14	207.11
30	4.81	99.24
40	2.6	61.41
50	1.56	43.81
60	1	34.11
70	0.64	28.07
80	0.39	23.81

## Data Availability

Not applicable.
